# The relationship between allergic status and adenotonsillar regrowth: a retrospective research on children after adenotonsillectomy

**DOI:** 10.1038/srep46615

**Published:** 2017-04-18

**Authors:** Zirong Huo, Jun Shi, Yilai Shu, Mingliang Xiang, Jingrong Lu, Hao Wu

**Affiliations:** 1 Department of Otolaryngology-Head & Neck Surgery, Xinhua Hospital, Shanghai Jiaotong University School of Medicine, Shanghai, China; 2 Ear Institute, Shanghai Jiaotong University School of Medicine, Shanghai, China; 3 Shanghai Key Laboratory of Translational Medicine on Ear and Nose diseases (14DZ2260300), Shanghai, China; 4 Department of Otolaryngology–Head and Neck Surgery, Eye and ENT Hospital, Shanghai Medical College, Fudan University, Shanghai, China; 5 Key Laboratory of Hearing Medicine, National Health and Family Planning Commission, Shanghai, China; 6 Department of Otolaryngology-Head & Neck Surgery, Shanghai Ninth People’s Hospital, Shanghai Jiaotong University School of Medicine, Shanghai, China.

## Abstract

Adenotonsillar regrowth in children after adenotonsillectomy (T&A) for obstructive sleep apnea (OSA) is often seen in clinical treatment, however, the relationship between allergic disease and adenotonsillar regrowth remains unclear. In this retrospective study, children were assigned to either the recurrence or control group, and subdivided by age at operation. Among children over 36 months, those in the recurrence group had more allergic disease and higher IgE, IL-4, and IL-5 levels than the same-aged children in control group. The Paediatric Allergic Disease Quality of Life Questionnaire (PADQLQ) scores for nasal symptoms and activity were higher in children older than 36 months in recurrence group. The results of immunohistochemistry and immunofluorescence showed that FoxP3+ cells (Tregs) were less, while GATA3+ cells (Th2 cells) were more in recurrence group for all ages. Allergic status and low levels of FoxP3 were proved as independent risk factors for adenotonsillar regrowth by multivariate logistic regression. These results indicate that allergic disease is a risk factor for adenotonsillar regrowth in children following T&A for OSA, and this risk increases with age. The decreased level of Tregs and subsequent changes in immune function play an important role in the pathogenesis of adenotonsillar regrowth.

Obstructive sleep apnea (OSA) is a common heath problem affecting 1 to 3% of children during the first decade of life^1^. It is characterized by prolonged partial upper airway obstruction and/or intermittent complete obstruction; disrupts normal ventilation during sleep and normal sleep patterns; and is associated with neurocognitive, behavioral and cardiovascular morbidities^2^,^3^,^4^. Adenotonsillar hypertrophy has been recognized as a risk factor for OSA in children^5^, therefore adenotonsillectomy (T&A) has been the first-line treatment for pediatric OSA^6^.

Although T&A results in significant improvement for children with OSA, symptoms have been estimated to reoccur in approximately 20–30% of children after surgery, among which nearly 50% of these cases due to adenotonsillar regrowth^7^,^8^. Some clinical researchers have implied that children with asthma and/or allergic rhinitis (AR) are more likely to experience recurrence of OSA resulting in a decrease in long-term quality of life after T&A^9^,^10^. In addition, most children with tonsillar regrowth after intra capsular tonsillectomy have an upper respiratory tract allergy, supporting the belief that allergic disease may be associated with adenotonsillar regrowth^7^,^11^. However, few studies have explored the difference in the allergic status of children with or without adenotonsillar regrowth. The degree of correlation between allergies and adenotonsillar regrowth, as well as the possible mechanisms of tissue regrowth remain unclear.

In this article, a retrospective study was conducted, and the allergic status of children with or without adenotonsillar regrowth after T&A were investigated. We further explored the difference between subgroups divided according to age at operation.

## Results

### General information and clinical data

The general data of patients were shown in Table 1, and the details of different age groups were further shown in Supplementary Table S1. As expected, there was no significant difference in the male/female ratio or the age at first operation between the recurrence and control groups. The duration of snoring and mouth breathing for the recurrence group was not significantly different with the control group either. The BMI z-scores, data for the size of the hypertrophic tonsils, preoperative PSG results (AHI and lowest oxygen saturation), and the rate of extracapsular tonsillectomy showed no significant differences between two groups, either.

### Allergic status based on medical history, immunity indicators in serum, and PADQLQ

Data from the assessments of allergic status including the patients’ medical history, immune parameters in serum, PADQLQ results are displayed in Table 2, and the details of different age groups were further shown in Supplementary Table S2. Generally speaking, there were more patients with AR or asthma in the recurrence group compared with their control group peers. The difference in the number of patients with AR was statistically significant in children older than 36 months. More children aged between 36 and 72 months in the recurrence group had asthma.

Overall, the level of total serum IgE, IL-4, and IL-5 were much higher for the recurrence group compared with the control group (Table 2). Most of these differences lay in patients older than 36 months. And the differences in IgE and IL-5 between recurrence and control cases were negligible for the youngest children (Supplementary Table S2). The level of IFN-γ and IL-12 showed no significant differences between two groups.

Twenty-six questions in each PADQLQ were classified into four different domains (nasal symptoms, “other” symptoms, emotions, and everyday activities). For patients older than 36 month, nasal symptoms were more severe, and everyday activities were affected more in the recurrence than the control group, respectively (Supplementary Table S2). For patients older than 72 months, emotional problems were more severe in the recurrence group than the control group, respectively. The severity of symptoms in the other domains were similar between the two groups for the different ages.

### Immunoreactivity of adenotonsillar tissue

**Figure 1:**
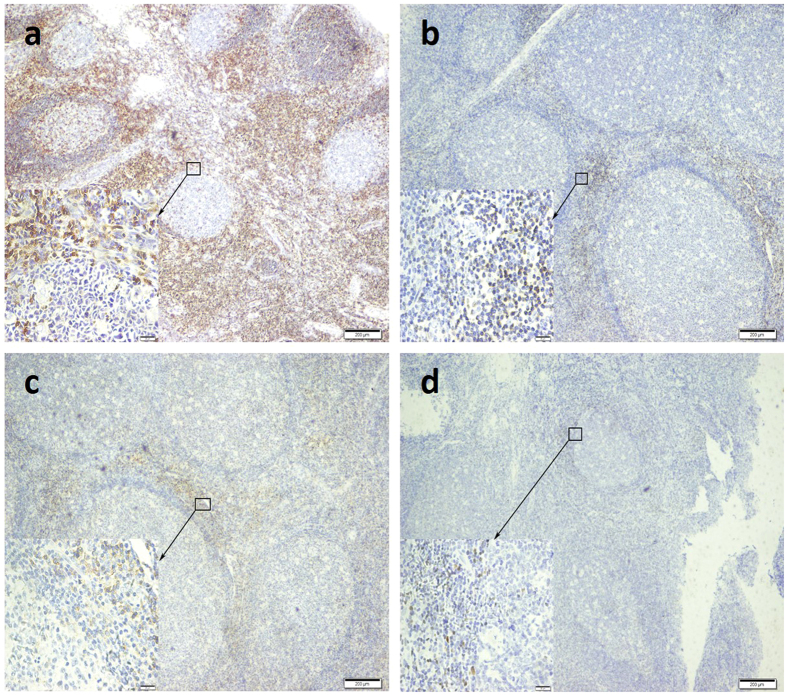
Localization of immunola belings of lymphocytes in adenotonsillar tissue. CD45 was extensively expressed in the extrafollicular areas, tonsillar mantle zones, and germinal centers (**a**). CD3+ and CD4+ lymphocytes were localized primarily in the extrafollicular areas (**b** and **c**). The percentage of FoxP3+ lymphocytes, also located in the extrafollicular areas, was relatively low (**d**). Magnification ×40 HPF in the large map and ×400 HPF in the small map. Scaling bars indicate 200 μm for maps of 40 HPF and 20 μm for maps of 400 HPF.

Immunohistochemistry and immunofluorescence were used to assess the proliferation and location of different lymphocyte subtypes in adenotonsillar tissue. Figure 1 shows the location of the subtypes. CD45 was extensively expressed in the extrafollicular areas, tonsillar mantle zones, and germinal centers; whereas, CD3+, CD4+ and FoxP3+ lymphocytes were primarily located in the tonsillar extrafollicular areas. GATA3 was rarely detected in the adenotonsillar tissue.

**Figure 2:**
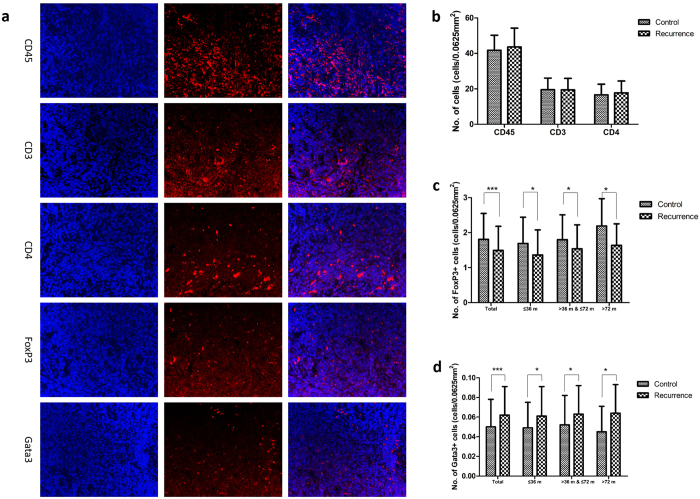
The number of CD45+, CD3+, CD4+, Gata3+ and FoxP3+ cells in the recurrence and control groups. (**a**) Representative fluorescent staining of CD45, CD3, CD4, Gata3 and FoxP3 in the adenotonsillar tissues of control group and recurrence group (Magnification ×200 HPF). (**b**) There were no significant differences in the number of CD45+, CD3+ and CD4+ cells between the recurrence and control groups. (**c**) The number of Gata3+ cells was much higher (p < 0.001) in the recurrence group compared with the control group. (**d**) The number of FoxP3+ cells was significantly lower in the recurrence group compared with the control group (p < 0.001). *p < 0.05; ***p < 0.01.

Overall, there was no significant difference between the recurrence and control groups in the number of total leukocytes (CD45+ cells) or T-lymphocytes (CD3+ and CD4+ cells). The number of FoxP3+ cells in the adenotonsillar tissue of the recurrence group was substantially lower than that in the control group (1.49 ± 0.69 vs. 1.81 ± 0.74, p < 0.001), suggesting that there were fewer Tregs in the recurrence group. On the contrary, there were much more Gata3+ cells (Th2-type cells) in the adenotonsillar tissue of the recurrence group compared with their control peers (0.062 ± 0.029 vs. 0.050 ± 0.028, p < 0.001). Comparisons were also made between the different age-matched subgroups as shown in Fig. 2.

### Logistic regression analysis

The results of logistic regression are shown in Table 3. Allergic disease is a risk factor for reoperation due to adenotonsillar regrowth. High level of IgE and IL-4 in the serum, and low level of FoxP3 expression are also associated with recurrence. In addition, the influence of allergic status and expression level of FoxP3 on reoperation was independent of other confounding factors, such as AHI, patient BMI, and Gata3 expression level in adenotonsilar tissues.

## Discussion

T&A has been the first line of treatment for children with OSA; however, 20–30% of children may not experience long-term resolution of OSA after surgery, and nearly half of these treatment failures are due to adenotonsillar regrowth^7^,^12^. McColley *et al*.^13^ showed that children with positive allergy tests were more likely to have OSA. One possible explanation is that allergy can lead to lymphoid hypertrophy, increasing the size of the tonsils and adenoids^14^. Therefore, we hypothesized that allergic disease might also be related to adenotonsillar regrowth after T&A, and our study demonstrates, for the first time, that an allergic diagnosis is a risk factor for adenotonsillar regrowth. In this study, allergic status was assessed by the medical history, total serum IgE, IL-4, IL-5, IL-12, and IFN-γ level, PADQLQ survey results, and immunohistochemistry findings for different types of lymphocytes. In addition, subgrouping patients according to age showed that the association between allergies and regrowth of adenotonsillar tissue is more significant in older children.

Previous studies have estimated that the prevalence of AR in children ranges from 10% to 40%^15^,^16^. In our study, 41.4% of children had a history of AR in the recurrence group, while this rate was 22.5% in the control group, who had no obvious adenotonsillar regrowth after T&A. Another study demonstrated that children with allergy are more likely to suffer from OSA^14^. In addition, the usage of montelukast and intranasal budesonide was found to be helpful for children with residual OSA after surgery, suggesting that treatment of AR and control of allergy would improve the outcome of adenotonsillectomy and avoid recurrence^17^.

Our study also showed that there were more patient complicated with asthma in the recurrence group compared with control group, especially for children between 36 and 72 months (Table 2 and Supplementary Table S2). Previous studies have explored the relationship between asthma and OSA, and demonstrated that the prevalence of OSA tended to increase in asthmatic children, with a breathing abnormality during Rapid-Eye-Movement (REM) sleep identified by PSG^18^,^19^,^20^. Also, OSA therapy seems to be helpful in reducing asthma symptoms^21^,^22^.

In our study, children in the recurrence group showed a significantly higher level of IgE, indicating that allergy may be one of the risk factors for postoperative recurrence due to adenotonsillar regrowth. Several theories on the pathophysiological link between OSA and AR have been proposed: (1) the chance of adenoid hypertrophy is greater in children with AR^23^; (2) patients with AR usually have an increase nasal resistance, which subsequently leads to upper airway obstruction^24^.

It has been revealed that OSA is associated with certain inflammatory cell profiles in patients with allergic disease^25^,^26^. To explore whether inflammatory cells also play a role in adenotonsillar regrowth, serial IHC was performed in our study to show the inflammatory cell profile. The number of total leukocytes (CD45+ cells), T-lymphocytes (CD3+ cells) as well as T-helper cells (CD4+ cells) were not significantly different between the recurrence and control groups, suggesting that recurrence of OSA due to adenotonsillar regrowth may not be related to a quantitative change in these lymphocytes.

The high level of total serum IgE with no obvious increase in specific types of lymphocytes turns our attention from a quantitative change to a possible functional change of lymphocytes. FoxP3 plays an important role in the development of and is a specific marker for the presence of CD4+ regulatory T cells (Tregs)^27^. This subset of CD4+ T cells that suppresses the functions of other lymphocytes, is essential in balancing immune responses, maintaining peripheral tolerance, and suppressing IL-4 production^28^. Current studies also suggest that defects in Tregs are involved in the pathogenesis of chronic inflammation, autoimmune disorders, and allergic diseases^29^,^30^,^31^. In allergic diseases, the decreased expression of Foxp3 may lead to a high level of IgE concentration and allow the population of Th2-type cells to expand, increasing the migration and activation of dendritic cells, eosinophils, and mast cells^27^,^32^. The Th2-type response is characterized by IgE-mediated degranulation and release of inflammatory mediators secreted by basophils and mast cells which, in turn, promote Th2-type cytokine production (IL-4, IL-5, and IL-13), leading to lymphocytic and eosinophilic infiltration into local tissues. In our study, the expression level of FoxP3 was much lower in the recurrence group, exactly the opposite to the level of Gata3 (Fig. 2). And the serum levels of IgE, IL-4 and IL-5 in recurrence group were subsequently higher compared with control patients (Table 2). Our study is the first to demonstrate that a decreased level of FoxP3, subsequent proliferation of Th2-type cells, and a subsequent imbalance of Th2/Th1 subtypes may be responsible for adenotonsillar regrowth.

It has been demonstrated that the prevalence of AR and asthma varies with age^33^. Therefore, we divided the patients into 3 subgroups according to their age and explored the allergic status within each age group. Subgroup analysis was used to identify the influence of age on the association between allergies and adenotonsillar tissue regrowth.

In the group of children older than 72 months, allergic status had a more significant effect on their prognosis of OSA after adenotonsillectomy. Statistically significant differences between the recurrence and control groups were found for allergic history, IgE concentration, serum levels of IL-4 and IL-5, effects on patients’ emotions and everyday activities based on the PADQLQ, and the number of Th2-type lymphocytes and FoxP3+ cells in adenotonsillar tissues. On the contrary, the influence of allergic status on surgical prognosis was similar between groups for children younger than 36 months. These findings may be explained by the prevalence of allergic diseases: 52% in adolescents aged between 13 and 14 years old^34^; 37% in children from 6 to 7 years old^35^; and only 8.5 to 12.2% in younger children^13^. As the prevalence of allergic diseases increases with age, the impact of allergy on the surgical prognosis of OSA becomes more significant. Therefore, anti-inflammatory medications, such as nasal corticosteroid and montelukast, should be considered after T&A, especially for older children.

Many other factors may influence the outcome of T&A, such as the severity of OSA (preoperative AHI), BMI, and early-onset infection^36^,^37^. To explore the possible risk factors which may lead to postoperative recurrence, multivariate logistic regression was applied. Preoperative AHI and BMI of the recurrence group compared with control group had no significant differences. This suggests that allergic status is an independent risk factor for adenotonsillar regrowth. Intracapsular and extracapsular tonsillectomy also had no obvious influence on recurrence, consistent with the findings by Eviatar E *et al*.^38^.

Limitations of this study include the retrospective nature and small sample size, especially when patients were divided into age-matched subgroups for analysis. The prospective trial with a larger population is necessary to make a more credible conclusion. In addition, PADQLQ was based on parental answers, which may be subjective. To reduce bias, IgE concentration was measured in all patients as an objective measure of allergic status. Finally, we collected only the patients who achieved postoperative follow-up more than 5 years in our clinic. Therefore, the miss of patients without regular follow-up may bring bias to this study.

In conclusion, allergic disease is associated with adenotonsillar regrowth in children with OSA after T&A independent of AHI and BMI. In addition, this negative influence is more significant with increasing age. The decreased level of Tregs (Foxp3+ cells) and the subsequent imbalance of Th2/Th1 may play an important role in the development of adenotonsillar regrowth and recurrent OSA. Treatment of allergic disease may prevent recurrent OSA after T&A, especially for older children.

## Methods

### Data collection

Retrospective data of children with sleep problems related to OSA were collected from the Department of Otolaryngology Head and Neck Surgery, Xinhua Hospital and Eye and ENT Hospital between January 2005 and December 2011. We focused on the children diagnosed with OSA due to adenotonsillar hypertrophy and underwent T&A. After operation, each patient went to the clinic once a month during the first half year after operation, and then once every 3 months for the first year, and once every half year thereafter till the 5^th^ year. The results of pharyngeal examination and fiber endoscopy were recorded at each follow-up visit. Tonsillar size was graded from 0 to 4+ and tonsillar hypertrophy was defined as grade 3+ or 4+ 39. Adenoidal hypertrophy was diagnosed on fiber endoscopy. The adenoid tissue extending to over 50% of the rhinopharynx was regarded as adenoidal hypertrophy.

According to this criteria, 116 children were diagnosed as adenotonsillar hypertrophy within 5 years after T&A surgery, and selected to recurrence group. Using the same follow-up strategy, 178 sex- and age-matched children without adenotonsillar regrowth evidence in their follow-up were enrolled in the control group. Cranio-facial anomalies, genetic and neuromuscular diseases, and cognitive deficits were excluded in our study.

Because it has been demonstrated that the prevalence of AR and asthma varies according to age^33^, we divided each group into three subgroups according to their age at operation (≤36 months, >36 months and ≤72 months, and >72 months). A general survey focusing on demographic data, clinical symptoms before operation, and physical examination findings was performed for each group. Body mass index (BMI) z-score was evaluated according to age and gender by an established guideline^40^.

Ethical approval for this study was given by the Ethical Committee of Xinhua Hospital and Eye and ENT Hospital, and the informed consent was obtained from all parents of the children in our study. All methods were performed in accordance with the relevant guidelines and regulations.

### Polysomnography and adenotonsillectomy

Each child underwent a fully-attended overnight polysomnography (PSG; Compumedics Company, Australia) in our department before operation. The recorded data included ECG, nose and mouth breathing, chest and abdomen movement, heart rate, pulse, and oxygen saturation. All of children in our study met specific criteria for T&A: (1) adenotonsillar hypertrophy, and (2) symptoms related to OSA > 3 nights/week, and (3) apnea/hypopnea index (AHI) indicative of >5 episodes/hour. Tonsillectomy was performed using a coblation method, while adenoidectomy was performed using a microdebrider-assisted method. Both intracapsular tonsillectomy (i.e. tonsillotomy) and extracapsular (i.e. traditional) tonsillectomy^41^ were acceptable for tonsillar resection in our series. All surgical procedures were performed in a single-stage by the same surgical team to ensure consistency of treatment strategies.

### Total serum IgE and Paediatric Allergic Disease Quality of Life Questionnaire

For each patient, total serum IgE, IL-4, IL-5, IL-12, and IFN-γ were tested by Elisa before surgery (peripheral venous blood sample; ImmunoCAP^®^ assay).

The effect of allergic status on a child’s daily life was retrospectively assessed using results from the Paediatric Allergic Disease Quality of Life Questionnaires (PADQLQs)^42^, which includes questions about patient symptoms (5 nasal and 9 “other”), emotions (4), and everyday activities (8). The 26 items are scored using a 7-point ordinal scale (0–6) to assess the influence of specific symptoms on a child’s daily life as recommended^42^. The scores on each item are summed to make a total score (0 to 156). Questionnaires had been completed by the parents or caregivers prior to surgery.

### Immunohistochemistry and immunofluorescence of adenotonsillar tissues

Immunohistochemistry and immunofluorescence were used to assess the number of total leukocytes (CD45+ cells), T-lymphocytes (CD3+ cells), T-helper cells (CD4+ cells), Th2-type (GATA-binding protein 3 [Gata3+]) cells, and Tregs (factor forkhead box P3 positive [FoxP3+]) cells.

Immunohistochemistry of the tumor tissue sections was performed as described previously^43^. Briefly, the 2-μm sections were dewaxed, rehydrated, and rinsed with PBS (pH 7.4). An antigen-demarking procedure was carried out following high temperature heating, immersion in Tris/EDTA buffer (10 mM Tris, 1 mM EDTA, 0.05% Tween-20, pH 9.0), and rinsing with PBS. Endogenous peroxidases were blocked with 3% H2O2 for 8 minutes, and nonspecific binding was blocked with 5% normal goat serum for 1 hour. Sections were then serially incubated with anti-CD4 antibody (1:30, GT214029, Gene Tech, Shanghai, China), anti-CD3 antibody (NCL-L-CD3-565, Lot # 6030008, Novocastra, Leica Biosystems, Newcastle, UK), anti-CD45 (1 μg/ml; MEM-28, ab8216, Abcam, Cambridge, UK), anti-GATA3 (1:50; GT218729, Gene Tech, Shanghai, China), anti-FoxP3 (5 μg/ml; 236 A/E7, ab20034, Abcam, Cambridge, UK) at 4 °C for 24 h, and then washed in PBS. Next, sections were incubated for 30 min in goat anti-rabbit/mouse IgG-HRP polymer, and then incubated with DAB Chromogen for 2 min. Finally, sections were photographed under a light microscope (×200 magnification).

Immunofluorescence was performed with the same anti-CD3, CD4, CD45, FoxP3, and GATA3 antibodies, and then incubated in for 30 min in fluorescence secondary antibodies as described previously^44^. Sections were observed under a fluorescence microscope (×200 magnification).

### Statistical analysis

Statistical analyses were performed using SPSS software (version 23.0; SPPS Inc., Chicago, IL). Normally distributed continuous data for the two groups were compared with the Student’s t-test, while comparison of non-normally distributed continuous data required the Mann-Whitney U test. The Chi-square test was used to compare categorical data. Then, a multivariate logistic regression model was built to eliminate possible confounding factors.

## Additional Information

**How to cite this article:** Huo, Z. *et al*. The relationship between allergic status and adenotonsillar regrowth: a retrospective research on children after adenotonsillectomy. *Sci. Rep.* **7**, 46615; doi: 10.1038/srep46615 (2017).

**Publisher's note:** Springer Nature remains neutral with regard to jurisdictional claims in published maps and institutional affiliations.

## Supplementary Material

Supplementary Information

## Figures and Tables

**Table 1:** Comparison of general data between recurrence and control group.

	Recurrence group N = 116	Control group N = 178	p value
Gender			
Male	67 (57.8%)	98 (55.1%)	NS
Female	49 (42.2%)	80 (44.9%)	
Age at operation (m)	49.83 ± 22.23 (11–114)	53.03 ± 26.08 (13–144)	NS
Duration of snoring and mouth breathing (m)	14.07 ± 8.60 (2–48)	13.71 ± 10.40 (2–60)	NS
MBI z-score before surgery	0.76 ± 1.03	0.74 ± 1.08	NS
Size of tonsil before operation			
3+	66 (56.9%)	111 (62.4%)	NS
4+	50 (43.1%)	67 (37.6%)	
Preoperative PSG			
AHI	12.4 ± 2.8	12.0 ± 3.1	NS
Lowest SaO_2_%	85.6 ± 2.8	85.1 ± 2.8	NS
Extracapsular tonsillectomy	26 (26.7%)	32 (28.1%)	NS

Continuous variables are presented as mean ± SD, while categorical variables as frequency (percentage).

BMI: body mass index; PSG: polysomnography; AHI: apnea/hypopnea index; RDI: respiratory distress index.

**Table 2:** Allergic status based on medical history, laboratory tests and PRQLQ results.

	Recurrence group N = 116	Control group N = 178	p value
Medical history			
AR	48 (41.4%)	40 (22.5%)	0.001*
asthma	16 (13.8%)	11 (6.2%)	0.045*
IgE (KU/L)	94.29 ± 77.56	71.31 ± 41.60	0.004*
IFN-γ (pg/ml)	50.60 ± 10.94	51.61 ± 9.50	NS
IL-12 (pg/ml)	35.96 ± 7.86	35.15 ± 7.33	NS
IL-4 (pg/ml)	63.03 ± 16.22	49.51 ± 13.06	<0.001*
IL-5 (pg/ml)	33.28 ± 8.81	28.63 ± 8.91	<0.001*
PADQLQ			
Nose symptoms	16.64 ± 6.53	15.36 ± 5.37	NS
“Other” symptoms^a^	6.34 ± 2.85	6.06 ± 2.56	NS
Emotions	9.03 ± 3.31	8.50 ± 3.01	NS
Everyday activities	16.38 ± 10.94	10.96 ± 8.74	NS

Continuous variables are shown as mean ± SD, and categorical variables are shown as frequency (percentage).

IL: interleukin; IFN: interferon; PADQLQ; Paediatric Allergic Disease Quality of Life Questionnaire; AR: allergic rhinitis.

a “Other” symptoms include eyes, ears, lungs and skin symptoms^16^.

*p < 0.05.

**Table 3:** Logistic regression analysis of possible risk factors for adenotonsillar regrowth.

Variables	OR	Lower 95% confidence limit for OR	Upper 95% confidence limit for OR	p value
AHI	0.928	0.845	1.019	0.118
BMI z-score	1.150	0.870	1.519	0.327
Allergic disease	2.953	1.614	5.401	<0.001*
IgE	0.988	0.982	0.993	<0.001*
IL-4	0.937	0.918	0.956	<0.001*
FoxP3	2.180	1.410	3.369	<0.001*
GATA3	0.001	<0.001	9.559	0.131

BMI: body mass index; IgE: immunoglobulin E; IL: interleukin; FoxP3: factor forkhead box P3; GATA3: GATA-binding protein 3; OR: odds ratio; CI: confidence interval.

*p < 0.05.
