# Supplementary material for: The relationship between allergic status and adenotonsillar regrowth: a retrospective research on children after adenotonsillectomy
Source: Sci Rep. 2017 Apr 18;7:46615. doi: 10.1038/srep46615 (PMC5394537; doi:10.1038/srep46615)
Supplement: Supplementary Information [file srep46615-s1.pdf]

The relationship between allergic status and adenotonsillar regrowth: a retrospective research on children after adenotonsillectomy

Zirong Huo[1,2,3]; Jun Shi[1,2,3], Yilai Shu[4,5], Mingliang Xiang[1,2,3], Jingrong Lu [1,2,3*], Hao Wu[2,3,6*]

[1] *Department of Otolaryngology-Head & Neck Surgery, Xinhua Hospital, Shanghai Jiaotong University School of Medicine;*

[2]*Ear Institute, Shanghai Jiaotong University School of Medicine;*

[3]*Shanghai Key Laboratory of Translational Medicine on Ear and Nose diseases (14DZ2260300);*

[4]*Department of Otolaryngology-Head and Neck Surgery, Eye and ENT Hospital, Shanghai Medical College, Fudan University;*

[5]*Key Laboratory of Hearing Medicine, National Health and Family Planning Commission;*

[6]*Department of Otolaryngology-Head & Neck Surgery, Shanghai Ninth People's Hospital, Shanghai Jiaotong University School of Medicine.*

Supplementary Table S1. Comparison of general data between recurrence and control group in three different age groups

	Age[a]: ≤ 36 months			Age[a]: >36 months & ≤ 72 months			Age[a]: > 72 months		
	**Recurrence group N=42**	**Control group N=60**	**p value**	**Recurrence group N=57**	**Control group N=93**	**p value**	**Recurrence group N=17**	**Control group N=25**	**p value**
**Gender**									
**Male**	23 (54.8%)	32 (53.3%)	NS	34 (59.6%)	51 (54.8%)	NS	10 (58.8%)	15 (60.0%)	NS
**Female**	19 (45.2%)	28 (46.7%)		23 (40.4%)	42 (45.2%)		7 (41.2%)	10 (40.0%)	
**Age at operation (m)**	28.26 ± 6.18 (11-36)	29.47 ± 5.59 (13-36)	NS	53.68±9.29 (38-72)	55.40 ± 10.84 (36-72)	NS	90.18 ± 12.17 (73-114)	100.80±26.50 (73-144)	NS
**Duration of snoring and mouth breathing (m)**	11.48 ± 5.13 (2-24)	12.30 ± 5.74 (3-24)	NS	14.12±8.75 (2-48)	11.76 ± 8.82 (2-42)	NS	20.29 ± 11.69 (3-48)	24.36 ± 16.76 (3-60)	NS
**MBI z-score before surgery**	0.76±1.00	0.83±0.99	NS	0.76±1.07	0.64±1.13	NS	0.75±1.00	0.89±1.13	NS
**Size of tonsil before operation**									
**3+**	24 (57.1%)	38 (63.3%)	NS	33 (57.9%)	59 (63.4%)	NS	9 (52.9%)	14 (56.0%)	NS
**4+**	18 (42.9%)	22 (36.7%)		24 (42.1%)	34 (36.6%)		8 (47.1%)	11 (44.0%)	
**Preoperative PSG**									
**AHI**	12.2±3.0	11.7±3.2	NS	12.3±2.7	12.7±3.2	NS	13.0±3.0	12.7±3.1	NS
**Lowest SaO_2%**	86.8±2.4	86.4±2.9	NS	85.2±2.9	84.4±2.5	NS	84.5±2.7	84.7±2.4	NS
**Extracapsular tonsillectomy**	5 (11.9%)	5 (8.3%)	NS	15 (26.3%)	18 (19.4%)	NS	6 (35.3%)	9 (36.0%)	NS

Continuous variables are presented as mean ± SD, while categorical variables as frequency (percentage).

BMI: body mass index; PSG: polysomnography; AHI: apnea/hypopnea index; RDI: respiratory distress index.

a Age at the time of surgery

Supplementary Table S2. Allergic status based on medical history, laboratory tests and PRQLQ results in three different age groups

	Age[a]: ≤36 months			Age[a]: >36 months & ≤72 months			Age[a]: >72 months		
	**Recurrence group N=42**	**Control group N=60**	**p value**	**Recurrence group N=57**	**Control group N=93**	**p value**	**Recurrence group N=17**	**Control group N=25**	**p value**
**Medical history**									
**AR**	8 (19.0%)	7 (11.7%)	NS	27 (47.4%)	25 (26.9%)	0.010*	13 (76.5%)	8 (32.0%)	0.012*
**asthma**	3 (7.1%)	3 (5.0%)	NS	9 (15.8%)	5 (5.4%)	0.033*	4 (23.5%)	3 (12.0%)	NS
**IgE (KU/L)**	76.77±92.43	59.78±43.71	NS	93.08±51.70	78.02±97.14	0.011*	141.64±93.82	89.72±38.60	0.017*
**IFN-γ (pg/ml)**	59.18±7.68	60.16±7.30	NS	47.06±9.21	49.02±6.47	NS	41.23±9.06	40.74±6.47	NS
**IL-12 (pg/ml)**	38.59±7.25	39.86±6.91	NS	32.45±6.23	34.02±5.97	NS	26.07±7.36	28.06±5.40	NS
**IL-4 (pg/ml)**	67.52±13.79	51.59±12.89	＜0.001*	63.26±17.39	49.98±13.63	＜0.001*	51.16±11.92	42.76±8.91	0.013*
**IL-5 (pg/ml)**	38.09±9.32	35.41±8.33	NS	32.12±7.09	26.31±6.95	＜0.001*	25.27±5.08	21.00±5.78	0.018*
**PADQLQ**									
**Nose symptoms**	13.88±6.83	14.63±5.51	NS	17.89±5.94	15.87±5.27	0.031*	19.24±5.52	15.20±5.42	0.024*
**"Other" symptoms[b]**	6.29±3.18	5.28±2.71	NS	5.88±2.65	6.42±2.36	NS	8.06±2.02	6.60±2.58	NS
**Emotions**	7.42±3.10	8.02±3.27	NS	9.49±2.92	8.59±2.81	NS	11.41±3.26	9.32±3.00	0.038*
**Everyday activities**	9.31±3.48	10.40±3.22	NS	12.98±5.02	10.86±3.51	0.003*	16.18±6.84	12.48±3.97	0.032*

Continuous variables are shown as mean ± SD, and categorical variables are shown as frequency (percentage).

IL: interleukin; IFN: interferon; PADQLQ: Paediatric Allergic Disease Quality of Life Questionnaire; AR: allergic rhinitis.

a Age at the time of surgery

b "Other" symptoms include eyes, ears, lungs and skin symptoms[15]

* $P < 0.05$
